# Role of endothelial cell markers in prognosis of hepatocellular carcinoma: Integrating bioinformatics analysis and experimental validation

**DOI:** 10.1371/journal.pone.0331580

**Published:** 2025-09-16

**Authors:** Zhaobin He, Jianqiang Cao, Yongzhe Yu, Cheng Peng

**Affiliations:** 1 Department of Hepatobiliary Surgery, General Surgery, Qilu Hospital, Shandong University, Jinan, Shandong, China; 2 Department of Geriatric Medicine, Qilu Hospital of Shandong University, Jinan, Shandong Province, China; University of Helsinki: Helsingin Yliopisto, FINLAND

## Abstract

**Background:**

Hepatocellular carcinoma (HCC) is a highly prevalent malignancy with poor prognosis. Endothelial cells (ECs) play a crucial role in HCC progression, yet their involvement at the single-cell level remains underexplored. This study aimed to identify ECs-specific markers and develop a prognostic multi-gene signature for HCC using single-cell RNA sequencing (scRNA-seq).

**Materials and methods:**

Single-cell transcriptomic data from 12 HCC samples were analyzed to identify EC-associated genes. A prognostic gene signature was constructed using Lasso-Cox regression analysis based on The Cancer Genome Atlas (TCGA) cohort and subsequently validated using an independent cohort from the International Cancer Genome Consortium (ICGC). Immunohistochemistry (IHC) and Western blotting were employed to experimentally validate gene expression in tissue samples.

**Results:**

Five EC-specific genes—*NDRG1, HBEGF, FKBP1A, KLRB1*, and *FDPS*—were identified as prognostic markers. The resulting multi-gene signature effectively stratified patients into high- and low-risk groups, with significant differences in overall survival. Validation in the ICGC cohort confirmed the model’s predictive performance. IHC and Western blotting results further confirmed the elevated expression of these genes in HCC tissues.

**Conclusions:**

This study established an EC-related prognostic signature that accurately predicts HCC prognosis. The identified markers may aid early diagnosis and serve as potential therapeutic targets for HCC treatment.

## Introduction

Hepatocellular carcinoma (HCC) is among the most prevalent malignant tumors globally, with liver cancer accounting for approximately 906,000 new cases in 2020, most of which were HCC. It ranks as the third leading cause of cancer-related mortality worldwide and is associated with a poor prognosis, reflected in a five-year overall survival rate of only 18% [1,2]. The prognosis of HCC is intricately linked to staging, with the Barcelona liver cancer staging (BCLC) system categorizing HCC into very early (BCLC-0), early (BCLC-A), intermediate (BCLC-B), advanced (BCLC-C), and end-stage (BCLC-D). Median survival times vary by stage, with BCLC-0 and A exceeding five years, BCLC-B at 2.5 years, BCLC-C at 2 years, and BCLC-D at approximately 3 months [3]. Early diagnosis and intervention are thus crucial to improving clinical outcomes [4]. In addition to conventional clinical diagnostic approaches such as symptoms, signs, and imaging tests, specific HCC markers are indispensable for the early detection of HCC. Alpha-fetoprotein (AFP), identified in the 1960s, remains the most widely used serum biomarker for HCC [5]. Post-hepatectomy and liver transplantation, AFP levels predict tumor recurrence likelihood and correlate significantly with HCC mortality [6,7]. However, AFP testing yields only approximately 60% diagnostic accuracy, with 15% to 30% of patients with advanced HCC presenting with negative AFP results. Although alternative biomarkers—including microRNAs, protein induced by vitamin K absence-II (PIVKA-II), AFP-L3, and heat shock protein 90 (Hsp90)—have demonstrated some utility in identifying AFP-negative HCC, their performance remains suboptimal [8–10]. Thus, there is a pressing need to identify novel HCC biomarkers with enhanced sensitivity and specificity.

HCC, a hypervascular tumor with arterial blood supply, relies heavily on angiogenesis for growth, progression, and metastasis [11]. A central mediator of this process is vascular endothelial growth factor (VEGF), which exerts its effects through interactions with its receptors, such as Flt-1 and FLK-1/KDR [12]. VEGF has been identified as a key driver of tumor angiogenesis in HCC [13], with recent meta-analyses indicating that VEGF levels in both tissue and serum can serve as prognostic indicators of overall survival in affected individuals [14]. Liver sinusoidal endothelial cells (LSECs), a subset of non-parenchymal cells, account for approximately 15–20% of total liver cells, although they occupy only about 3% of the liver’s volume [15]. Functionally, LSECs play a role in endocytosis, antigen presentation, and leukocyte recruitment, thereby forming a selective filtration barrier that sustains immune homeostasis within the hepatic environment [16]. These immunomodulatory roles of LSECs profoundly influence the tumor microenvironment (TME) and facilitate the progression of both primary and metastatic liver malignancies [17]. A critical aspect of LSEC function involves the presentation of soluble antigens via major histocompatibility complex class I (MHCI) to CD8 ⁺ T cells. This interaction leads to T cell anergy, wherein CD8 ⁺ T cells enter a non-responsive state and lose their cytotoxic capacity against tumor cells or antigens [18,19]. Moreover, recent findings have demonstrated that the overexpression of programmed death ligand 1 (PD-L1) on LSECs further impedes CD8 ⁺ T cell activation. This mechanism contributes to immune evasion and is strongly associated with poor prognosis in HCC patients [20].

In recent years, the advent of single-cell sequencing (scRNA-seq) has significantly advanced our comprehension of the role played by liver endothelial cells (ECs) in the onset and progression of HCC. Within the liver, three distinct types of ECs exist: LSECs, vascular ECs, and lymphatic ECs, with LSECs constituting the majority [21]. Differential gene expression analyses have revealed significant transcriptional disparities between ECs in paracancerous liver tissue and those in normal liver, suggesting tumor-induced modifications in the stromal compartment. In HCC tumor tissues, ECs not only exhibit altered gene expression patterns but also form distinct cell clusters. Notably, an increased proportion of arterial and lymphatic ECs has been observed in tumor samples, consistent with the augmented arterial supply characteristic of early-stage HCC [22].

In summary, while numerous studies have established the crucial role of ECs in HCC development and prognosis, investigations at the single-cell level remain limited. To our knowledge, no prior study has comprehensively examined the impact of ECs and their associated markers on HCC prognosis using scRNA-seq data. This study addresses that gap by systematically analyzing HCC scRNA-seq data to identify a set of EC-specific marker genes. These genes were subsequently employed to construct a multi-gene prognostic signature using data from The Cancer Genome Atlas (TCGA), with external validation conducted through the International Cancer Genome Consortium (ICGC). The expression of these genes in HCC was further verified via immunohistochemical (IHC) staining and Western blotting.

## Materials and methods

### Data acquisition from databases

We obtained the following datasets from publicly available repositories: 1) scRNA-seq raw data for twelve HCC samples from the Gene Expression Omnibus (GEO) (https://www.ncbi.nlm.nih.gov/gds); 2) gene expression files and clinical data for 376 HCC samples from the TCGA database (https://portal.gdc.cancer.gov/repository); 3) gene expression files and clinical data for 260 patients with HCC from the ICGC database (https://dcc.icgc.org/releases/current/Projects/).

### Processing of scRNA-seq data

The Seurat package in R was utilized to process the single-cell RNA sequencing (scRNA-seq) data. Correlation analyses were conducted to assess the relationships among sequencing depth, the percentage of mitochondrial gene expression, and the total number of genes detected. Cells expressing fewer than 50 genes or with mitochondrial gene content exceeding 5% were excluded from further analysis. The scRNA-seq data were normalized using the LogNormalize method, after which the top 1,500 highly variable genes were identified through variance analysis. Dimensionality reduction was performed using principal component analysis (PCA) [23]. A cluster classification analysis using the t-SNE algorithm, with a cutoff threshold of logFC > 1 and an adjusted p-value of 0.05, was then conducted. A heatmap showcasing the top 10% important marker genes was generated [24]. Clusters were annotated using the SingleR package based on marker genes, and HCC cells were categorized into subsets using pseudotime and trajectory analyses via the monocle package [25]. Differentially expressed genes (DEGs) between these subsets, meeting the criteria of |log₂(fold change)| > 1 and a false discovery rate (FDR) < 0.05, were identified as differentiation-related genes (DRGs). From this analysis, a subset of EC-associated marker genes was subsequently determined.

### Construction and validation of the ECs-related prognostic multi-gene signature

Gene expression data were first normalized, and differentially expressed genes (DEGs) between tumor and adjacent non-tumorous tissues were identified using the limma package in R, applying a false discovery rate (FDR) threshold of < 0.05. Univariate Cox regression analysis was then conducted to screen for EC–related genes significantly associated with overall survival (OS), using an adjusted p-value < 0.001 as the selection criterion. The intersection of DEGs and OS-associated EC-related genes was used to derive a refined gene set, which was visualized with a Venn diagram. To construct a prognostic model, least absolute shrinkage and selection operator (Lasso) Cox regression was applied for variable selection and dimensionality reduction, utilizing the glmnet and survival R packages. The optimal penalty parameter (λ) was identified through 10-fold cross-validation based on the minimum criteria [26]. A prognostic risk score for each patient was calculated using the weighted expression of selected genes, and patients were subsequently stratified into high-risk and low-risk groups based on the median risk score. PCA and t-SNE were conducted to examine the distribution of the two risk groups. Survival analysis and receiver operating characteristic (ROC) analysis were performed to evaluate the accuracy of the signature in predicting patient prognosis using the “survival” and “timeROC” R packages, respectively.

### Human tissue specimens

A total of 7 hepatic carcinoma specimens, 1 adjacent non-tumorous tissue sample and 1 liver biopsy sample from normal individuals, acquired randomly between December 2021 and April 2022, were selected from Qilu Hospital of Shandong University, located in Jinan, Shandong Province, China. These specimens were either immediately frozen at −80°C or underwent formalin fixation and paraffin embedding for preservation. The study was conducted in accordance with the Declaration of Helsinki, and approved by The Ethics Committee of Qilu Hospital, Shandong University (protocol code: KYLL-2020(KS)-555 and date of approval: March 25, 2020). Informed written consent was obtained from all subjects involved in the study.

### Cell lines and culture conditions

Human umbilical vein endothelial cells (HUVECs) (PCS-100–013) and human hepatic carcinoma cells (HepG2) (HB-8065) were procured from the American Type Culture Collection (Gaithersburg, MD, USA). Upon resuscitation, the HUVECs were cultured in DMEM complete medium with 15% FBS in a constant temperature cell incubator at 37 °C and 5% CO_2_. The medium was refreshed every 1–2 days. Upon reaching approximately 80% confluence, the culture medium was aspirated, and cells were digested using 0.25% trypsin. Subculturing was performed at a ratio of 1:3. HepG2 cells were maintained in MEM supplemented with 10% FBS, 100 U/mL penicillin, and 100 mg/mL streptomycin, and incubated at 37 °C in a humidified atmosphere containing 5% CO₂ for 48 h. After this period, the culture medium was removed, and the cells were washed three times with phosphate-buffered saline (PBS). A serum-free medium was then added to induce overnight starvation. On the following day, the supernatant from the HepG2 cell culture was collected, centrifuged at 2,000 rpm for 5 min, and filtered through a 0.22 µm microporous membrane to remove residual floating tumor cells. The resulting solution was diluted to a 50% volume ratio and reserved for subsequent experiments.

### Antibodies and reagents

The Rabbit anti-human monoclonal antibody EPR4628 (ab109007), specifically targeting FDPS, and the Mouse anti-human monoclonal antibody 1E5-A12 (ab58072), directed against FKBP1A, were both procured from Abcam (Cambridge, MA, USA). Additionally, the Rabbit polyclonal antibody ab197979, directed against KLRB1 (CD161), was also obtained from Abcam. The following monoclonal antibodies, A16365 and A4050, which specifically target HB-EGF and NDRG1, respectively, were acquired from ABclonal (Wuhan, Hubei, China). Reagents for sodium dodecyl sulfate-polyacrylamide gel electrophoresis (SDS-PAGE), including molecular weight markers, were sourced from Bio-Rad Laboratories (Hercules, CA, USA).

### Immunohistochemistry

Immunohistochemistry analysis was performed on 5-μm tissue sections using monoclonal mouse antibodies specific to the respective target proteins. Tissue sections were first deparaffinized and rehydrated, followed by heat-induced epitope retrieval using Borg Decloaker high pH buffer in a Biocare Medical Decloaking Chamber. Endogenous peroxidase activity was quenched with 3% hydrogen peroxide. An avidin-biotin blocking kit was applied, after which the sections were incubated overnight at 4 °C with the primary antibody against integrin αvβ6 (dilution 1:500). Subsequent steps included treatment with streptavidin-horseradish peroxidase and Betazoid Diaminobenzidine for color development. Negative controls were prepared using an identical concentration of mouse immunoglobulin IgG1 (Dako). The following day, biotinylated anti-mouse IgG (1:200) was applied to the slides, followed by treatment with horseradish peroxidase (HRP)-labeled streptoantibiotin (Dako) for 15 minutes. Counterstaining was performed using Dako Hematoxylin, followed by rinsing with water, dehydration with alcohol and xylene, and coverslipping. All incubations were carried out at room temperature. Digital slide scanning and analysis were conducted using Case Viewer software (3DHistech, Hungary; Version 2.4), with appropriate positive and negative controls included throughout the procedure.

### Assessment of target protein expression in tissue sections

Staining evaluation for the target proteins was independently conducted by two experienced pathologists. In cases of discrepancy, a consensus was reached through joint review and discussion. The assessment comprised two components: the proportion of positively stained cells and the staining intensity. The proportion score reflected the estimated percentage of stained cells and was graded as follows: 0 (0%), 1 (<20%), 2 (20–50%), 3 (51–75%), and 4 (>75%). Staining intensity was evaluated on a scale from 0 to 3: 0 (no staining), 1 (weak staining, pale brown), 2 (moderate staining, brown), and 3 (strong staining, dark brown). To provide a semi-quantitative estimate of antigen expression levels, the final score for each section was calculated as the sum of the proportion and intensity scores. Based on the final score, staining expression was classified as follows: a score <2 indicated negative expression, scores of 2–4 indicated low (weak) expression, and scores ≥5 indicated high (strong) expression. For analytical purposes, both low and high expression levels were considered positive for the target protein [27,28].

### Western blotting

HUVECs were exposed to supernatant collected from the HepG2 culture medium for 48 hours, while the control group had their culture medium replaced with PBS. Both adherent and floating cells were collected and stored at –80°C. Proteins were extracted from the cells for concentration determination. Protein concentration was quantified using the BCA Protein Assay Reagent. Equal amounts of protein (10 μg) were loaded onto a 12.5% SDS-PAGE gel and electrophoresed under non-reducing conditions. After electrophoresis, the proteins were transferred to nitrocellulose membranes. Confirmation of equivalent protein loading in each lane was established by staining the nitrocellulose membranes with Ponceau-S Stain, using the 42 kDa β-actin band as a reference marker. Subsequently, the membranes were probed with primary antibodies against key signaling pathway factors, followed by peroxidase-conjugated secondary antibodies. Protein visualization was achieved using the Enhanced Chemiluminescence Detection System (Pierce, Rockford, Illinois, USA), following the manufacturer’s instructions. Optical density was analyzed using Image J software (National Institute of Health, USA).

### Statistical analysis

All statistical analyses were conducted using R language (version 4.1.1) software. Continuous variables were presented as the mean ± standard deviation (SD), and comparisons between groups were conducted using the Student’s t-test or analysis of variance (ANOVA), as appropriate. The chi-square (χ²) test was employed for the comparison of categorical variables. Survival outcomes were evaluated using the Kaplan–Meier method, with statistical differences assessed via the log-rank test. A p-value of less than 0.05 was considered indicative of statistical significance.

## Results

**Fig 1:**
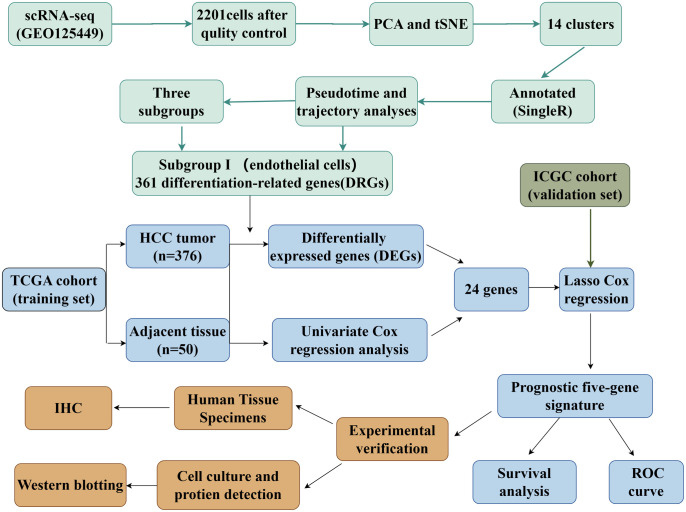
The flowchart of the study (by Figdraw).

The flowchart of the study is depicted in Fig 1.

### ECs-related genes were obtained by single-cell sequencing analysis

**Fig 2:**
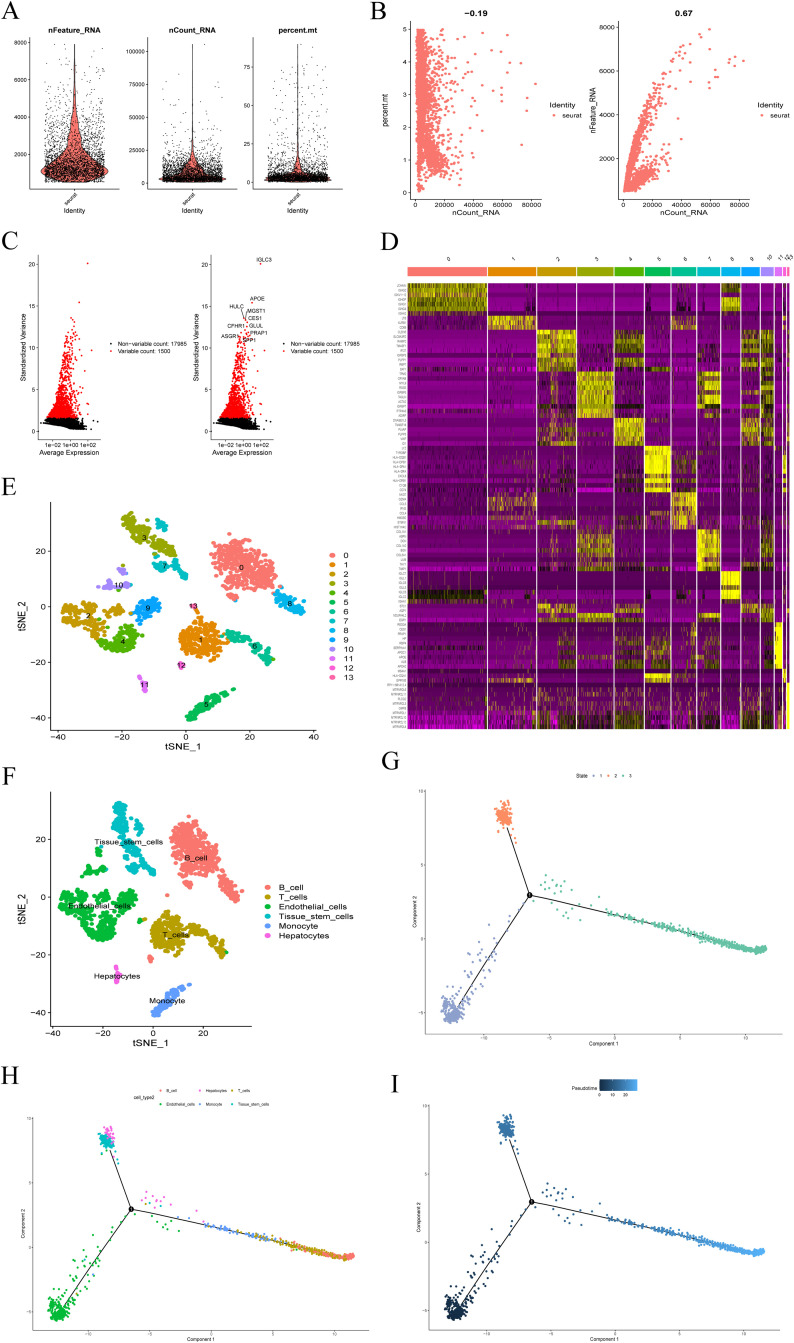
Processing and analysis of scRNA-seq data. (A) Following quality control and logarithmic normalization, 2201 cells from 12 HCC samples were utilized for subsequent analysis. (B) Correlation analysis revealed a negative correlation between sequencing depth (nCount_RNA) and the number of mitochondrial genes (percent.mt), while a positive correlation was observed with the total number of genes detected (nFeature_RNA). (C) A total of 17985 genes were included, with 1500 of the most differentially expressed genes selected. (D) After identifying and filtering differentially expressed genes (|log2(FC)| > 1, adjusted P value < 0.05), the top 10% were visualized in the heat map. (E) The 2201 HCC cells were segregated into 14 clusters. (F) Clusters were annotated based on marker genes. (G-I) Pseudotime and cell trajectory analysis classified all cells into three subgroups, with subgroup I predominantly composed of endothelial cells in the early stage of cell differentiation.

After quality control and normalization of the GSE125449 (Set1) dataset, 2,201 cells from 12 HCC samples were retained for subsequent analysis (Fig 2A). Correlation analysis revealed a negative correlation between sequencing depth and the number of mitochondrial genes and a positive correlation with the total number of detected genes (Fig 2B). A total of 17,985 genes were included, with 1,500 selected as the most differentially expressed genes (Fig 2C). PCA was employed to reduce the dimensionality of the 1,500 differentially expressed genes, selecting the first 15 principal components for further analysis. Differentially expressed genes were identified across clusters, and, following further filtering (|log2(FC)| > 1, adjusted P value < 0.05), the top 10% of these genes were visualized in the heat map (Fig 2D). Utilizing the tSNE algorithm, the 2,201 cells were classified into 14 clusters (Fig 2E). Annotation of these clusters was performed based on marker genes, revealing ECs in clusters 2, 4, 9, 10, and 13, monocytes in cluster 5, B cells in clusters 0, 8, and 12, hepatocytes in cluster 11, T cells in clusters 1 and 6, and tissue stem cells in clusters 3 and 7 (Fig 2F). Pseudotime and cell trajectory analysis divided all cells into three subgroups: subgroup I, predominantly comprising ECs in the early stage of cell differentiation; subgroup II, mainly containing hepatocytes and tissue stem cells; and subgroup III, mainly encompassing B cells, T cells, and monocytes (Fig 2G-I). The marker gene set for subgroup I, referred to as DRGs, was extracted for further analysis (S1 Table).

### Clinicopathological features of patients with HCC in TCGA and ICGC cohorts

In the TCGA cohort, data from 376 patients with hepatocellular carcinoma (HCC) were analyzed. The mean age was 59.5 ± 13.5 years, with 122 females (32.4%) and 254 males (67.6%). Comprehensive clinicopathological information, including tumor grade and stage, was available for all patients. The ICGC cohort consisted of 260 HCC patients, with a mean age of 67.4 ± 10.0 years, including 68 females (26.2%) and 192 males (73.8%). Tumor staging data were also available for this cohort (Table 1).

**Table 1:** Clinicopathological features of patients with hepatocellular carcinoma in TCGA and ICGC cohort.

	TCGA (N = 376)	ICGC (N = 260)
Age (year)	59.5 ± 13.5	67.4 ± 10.0
Sex (%)		
Female	122 (32.4%)	68 (26.2%)
Male	254 (67.6%)	192 (73.8%)
Grade(%)		
G1	55 (14.6%)	NA
G2	180 (47.9%)	NA
G3	123 (32.7%)	NA
G4	13 (3.5%)	NA
unknown	5 (1.3%)	NA
Stage (%)		
I	175 (46.5%)	40 (15.4%)
II	86 (22.9%)	117 (45.0%)
III	86 (22.9%)	80 (30.8%)
IV	5 (1.3%)	23 (8.8%)
unknown	24 (6.4%)	0(0.0%)

TCGA: The Cancer Genome Atlas database; ICGC: International Cancer Genome Consortium database; NA: Not Available.

### Identification of ECs-related predictive genes in the TCGA cohort

**Fig 3:**
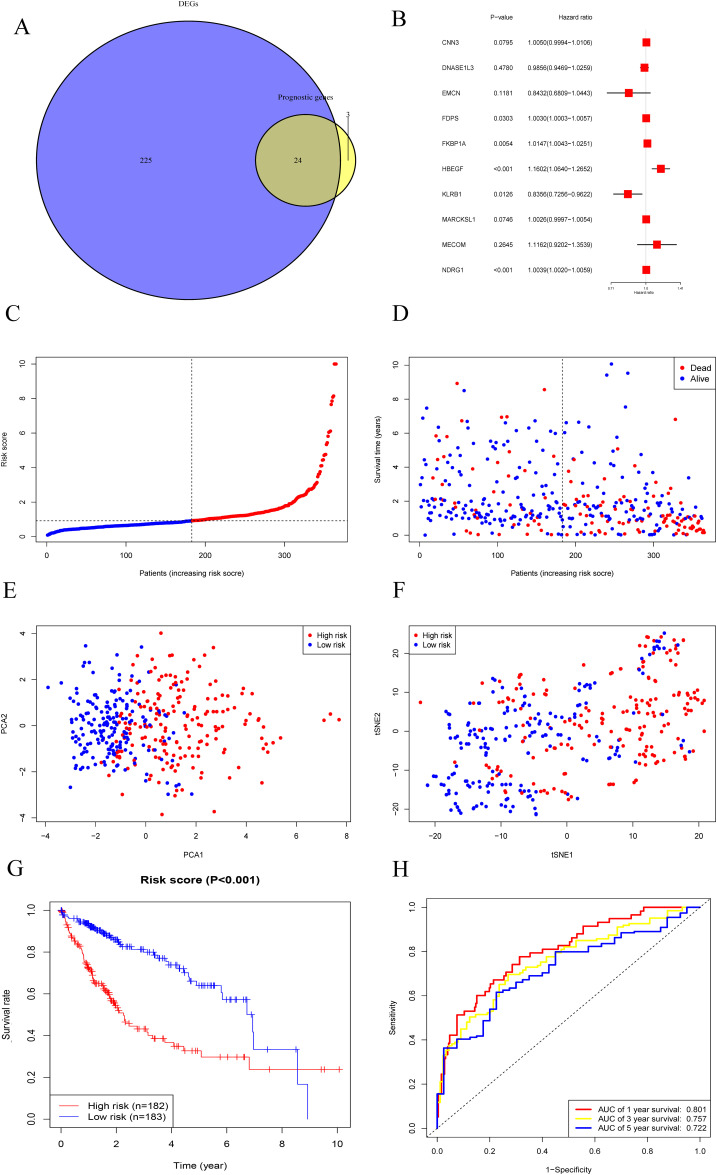
Establishment of multi-gene prediction signature in TCGA cohort. (A) A Venn diagram illustrated 249 DEGs and 27 genes with prognostic value, resulting in 24 ECs-related genes. (B) Forest plot showing five genes (NDRG1, HBEGF, FKBP1A, KLRB1, and FDPS) selected via Lasso-Cox and multivariable Cox regression analyses. (C) Risk stratification of patients with HCC into high- and low-risk groups based on the median risk score. (D–F) PCA and t-SNE analyses illustrating the distinct distribution of patients across the two risk groups, with shorter survival durations observed in the high-risk group. (G) Kaplan–Meier survival curves demonstrating significantly reduced overall survival in the high-risk group. (H) Time-dependent ROC curves evaluating the predictive performance of the model for 1-, 3-, and 5-year survival outcomes.

A total of 249 DEGs were identified between tumor and paracancerous tissues, constituting 69% of all ECs-related gene sets. Univariate Cox regression analysis identified 27 ECs-related genes with prognostic value. The intersection of these two groups yielded 24 genes (Fig 3A and S2 Table).

### Establishment of a five-gene predictive signature in the TCGA cohort

From the previously identified 24 genes, five genes (*NDRG1*, *HBEGF*, *FKBP1A*, *KLRB1*, and *FDPS*) were selected through Lasso-Cox regression analysis and multivariable Cox regression to construct a prognostic model (Fig 3B). Specifically, the regression coefficients (β values) for *NDRG1, HBEGF, FKBP1A, KLRB1*, and *FDPS* were 0.003941, 0.148613, 0.014574, −0.179648, and 0.002974, respectively. The risk score for each patient was calculated using the following formula: Risk score = (*NDRG1* expression * 0.003941) + (*HBEGF* expression * 0.148613) + (*FKBP1A* expression * 0.014574) + (*KLRB1* expression * −0.179648) + (*FDPS* expression * 0.002974). Patients were stratified into high-risk (n = 182) and low-risk (n = 183) groups based on the median risk score (median value: 0.9180203) (Fig 3C). PCA and t-SNE analyses demonstrated clear separation between the two groups, with the high-risk group exhibiting shorter survival durations (Fig 3D–F). Kaplan–Meier survival analysis revealed that overall survival was significantly reduced in the high-risk group compared to the low-risk group (Fig 3G). The predictive performance of the five-gene signature was further validated by time-dependent ROC curves, with AUCs of 0.801, 0.757, and 0.722 for 1-, 3-, and 5-year survival, respectively (Fig 3H).

### Verification of predictive signature in the ICGC cohort

**Fig 4:**
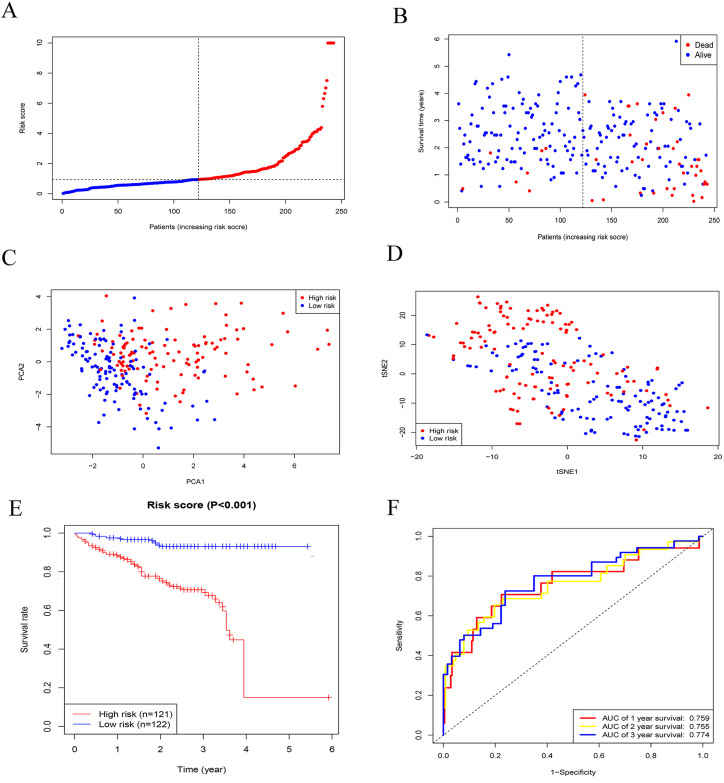
Verification of predictive signature in the ICGC cohort. (A) Patients with HCC were categorized into high and low-risk groups based on whether their risk scores exceeded the median. (B-D) A significant difference was observed in the distribution of patients between high-risk and low-risk groups. (E) The overall survival rate in the high-risk group was substantially lower than that in the low-risk group. (F) The area under the ROC curve (AUC) was calculated for predicting 1-, 2-, and 3-year survival rates.

To assess the robustness of the prognostic model developed from the TCGA cohort, the same risk score formula was applied to patients in the ICGC cohort. Based on the median risk score, patients were stratified into high-risk (n = 121) and low-risk (n = 122) groups (Fig 4A). PCA and tSNE analyses revealed significant differences in the distribution of patients between the high and low-risk groups (Fig 4B-D). Kaplan–Meier survival analysis showed that patients in the high-risk group had significantly poorer overall survival compared to those in the low-risk group (Fig 4E). The predictive performance of the model in the ICGC cohort was confirmed using ROC curves, with AUCs of 0.759, 0.755, and 0.774 for 1-, 2-, and 3-year survival, respectively (Fig 4F).

### Immunohistochemistry

**Fig 5:**
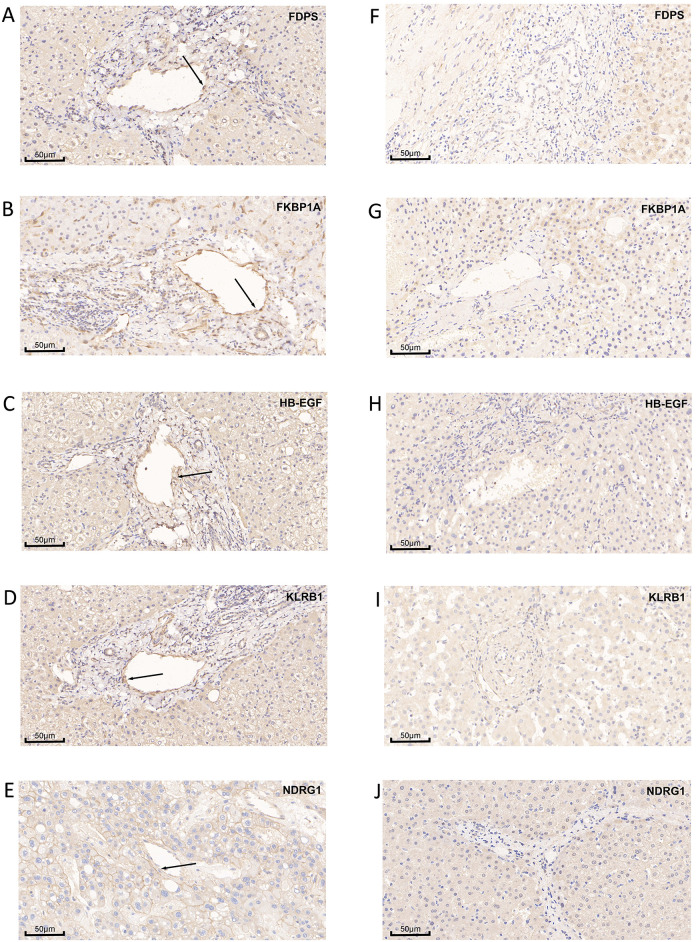
Immunohistochemical staining of target proteins in human hepatic cellular carcinoma and normal liver tissue. (A-E) Representative immunohistochemical images showing the expression of *FDPS, FKBP1A, HB-EGF, KLRB1* (*CD161*), and *NDRG1* in HCC tissue specimens. (F–J) Corresponding images of target protein expression in normal liver tissue. Positive staining was consistently observed in HCC samples, with notable localization to sinusoidal endothelial cells, whereas expression in normal liver tissue was largely negative or weakly positive.

The target proteins exhibited consistent expression patterns in liver samples, especially in vascular endothelial cells within the sinusoidal region. All seven HCC tissue specimens exhibited positive expression of the target proteins. Specifically, *FDPS* showed strong expression in all seven specimens. *FKBP1A* exhibited strong expression in two cases and weak expression in five. *HB-EGF* was strongly expressed in six cases and weakly in one. For *KLRB1* (*CD161*), strong expression was observed in four specimens and weak expression in three. *NDRG1* showed strong expression in five specimens and weak expression in two. In contrast, the normal liver tissue from a healthy control subject exhibited either negative or weakly positive expression for all five proteins. The most prominent differences in expression between HCC and normal tissues were observed in the ECs lining the hepatic sinusoids (Fig 5).

### Western blotting

**Fig 6:**
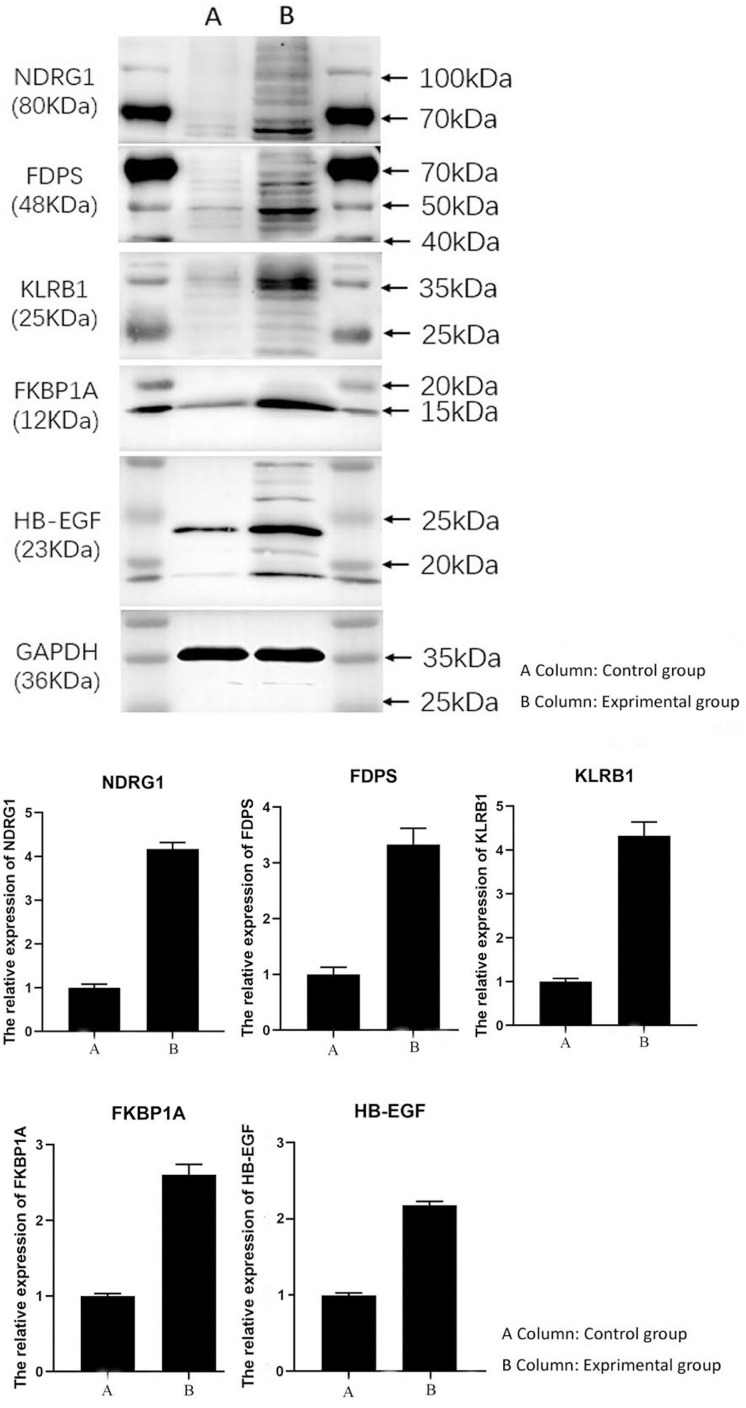
Western blotting images of target proteins expression level difference in experimental and control groups. The results demonstrated that the expression levels of all five target proteins in the experimental group were significantly higher than those in the control group.

To further investigate the differential expression of the target proteins between HCC and normal tissue, an in vitro simulation of the tumor microenvironment was performed. HUVECs were cultured and subsequently treated with the supernatant collected from HepG2 cell cultures to mimic the HCC microenvironment. In the control group, PBS was used in place of the HepG2 supernatant. Following incubation, total proteins were extracted from the HUVECs and subjected to Western blot analysis to evaluate the expression levels of the five target proteins. The results revealed significantly elevated expression levels of all five proteins—*FDPS, FKBP1A, HB-EGF, FKLRB1* (*CD161*), and *NDRG1*—in the experimental group compared to the control group. These findings were consistent with the immunohistochemistry results observed in clinical HCC specimens (Fig 6).

## Discussion

In this study, we conducted a comprehensive analysis to identify five ECs-related markers in HCC through an integrated approach combining bioinformatics analyses and experimental validation. Single-cell RNA sequencing data from HCC samples were obtained from the GEO database and processed using the R programming language. Fourteen distinct cellular clusters were identified and annotated as ECs, monocytes, B cells, hepatocytes, T cells, and tissue stem cells. Through pseudotime and trajectory analyses using the Monocle algorithm, all cells were categorized into three subgroups, with subgroup I—representing the early stage of cellular differentiation—predominantly composed of ECs. Genes derived from the EC-enriched trajectory were subjected to differential expression analysis, univariate Cox regression, and Lasso-Cox regression, ultimately leading to the identification of five key prognostic genes: *NDRG1, HBEGF, FKBP1A, KLRB1*, and *FDPS*. These genes were incorporated into a five-gene risk prediction signature based on data from the TCGA HCC cohort, which demonstrated strong prognostic accuracy. The predictive capability and robustness of this model were further validated using an independent cohort from the ICGC database. Experimental validation was conducted through immunohistochemical analysis, which confirmed positive expression of all five target proteins in HCC tissue specimens, with particularly high expression localized to LSECs. Additionally, under simulated in vivo conditions, HUVEC incubated in the culture supernatant of HepG2 showed significantly elevated expression levels of all five target proteins compared to the negative control group.

During the analysis of HCC scRNA-seq data, we employed the monocle algorithm, a commonly used time-series analysis tool in the R language, to transform cell differentiation states into gene expression sequences. This facilitated the sorting of single cells and depiction of the “trajectory” of cell differentiation. Our pseudotime and trajectory analyses revealed that the early phases of cell differentiation were predominantly composed of ECs, suggesting that ECs may play a critical role in the initiation and early progression of HCC. A comprehensive literature review indicated a lack of prior studies that systematically identified and validated EC-specific markers in HCC using scRNA-seq data in conjunction with the construction of a predictive risk signature. Therefore, the present study not only identifies novel EC-related markers but also introduces an innovative framework for investigating EC function in HCC at the single-cell level. Experimental validation supported these bioinformatic findings. When ECs were incubated with the culture supernatant of HCC cells, a significant upregulation in the expression of the five target proteins was observed, compared to the control group. This result indirectly demonstrates the influence of the HCC TME on ECs, consistent with the hypothesis that tumor-derived factors contribute to the reprogramming of EC behavior [29,30].

*NDRG1*, also known as Drd-1, Cap43, or RTP, is a homocysteine-responsive gene that can be induced in HUVECs by thiol reagents [31]. The literature on its expression in various cancers, such as gastric cancer, presents conflicting findings [32,33]. While some studies report decreased expression in tumor tissues compared to adjacent normal tissues [34–36], others suggest overexpression correlates with enhanced metastasis [37,38]. A study by Mei-Sze Chua et al. associated *NDRG1* overexpression with HCC differentiation degree, vascular invasion, and overall survival [39]. Jun Akiba et al. reported elevated *NDRG1* expression in HCC correlated with an increased rate of portal vein invasion, with high expression observed in surrounding invasive areas [40]. Heparin-binding epidermal growth factor (*HBEGF*), a member of the EGF family, plays a role in tissue growth, differentiation, blastocyst implantation, wound healing, smooth muscle cell proliferation, arteriosclerosis, and tumor growth [41]. Elevated *HBEGF* expression has been observed in HCC biopsy tissues relative to adjacent non-tumorous liver tissue [42]. *FKBP1A* (also known as *FKBP12*) initially characterized as an immunophilin that binds rapamycin and FK506, has been implicated in oncogenesis and targeted therapies [43,44]. However, its role in HCC remains underexplored, with most existing evidence derived from in silico analyses [45–47]. *KLRB1* (also known as *CD161*) encodes a type II transmembrane C-type lectin receptor expressed primarily on natural killer (NK) cells and subsets of CD4⁺ and CD8 ⁺ T cells [48]. It plays an essential role in immune cell differentiation, particularly in dendritic cells and monocytes [49]. Recent studies in tumor immunology-related prognostic models indicate that *KLRB1* is a crucial gene affecting the prognosis of lung adenocarcinoma, liver cancer, and breast cancer [50–52]. While *KLRB1* is typically downregulated in most tumors, high expression levels have been associated with a favorable prognosis [53]. Nonetheless, *KLRB1* expression has also been reported to increase in early recurrent HCC, possibly reflecting an altered cytotoxic immune state [54]. *FDPS*, a key enzyme in the mevalonate pathway, is overexpressed in various malignancies, including prostate cancer, glioblastoma, colon cancer, and pancreatic cancer [55–58]. Recent multi-gene prognostic signature studies have implicated *FDPS* in HCC pathogenesis as well [59,60]. Previous research has explored the roles of these tumor-derived factors in HCC development or prognosis, primarily at the protein, cellular, or bulk sequencing levels, providing insights at a relatively macroscopic scale. Leveraging single-cell sequencing tools allows us to identify these factors at the single-cell level, facilitating a more in-depth exploration of the precise molecular mechanisms driving tumor development and progression.

In this study, the five genes (*NDRG1, HBEGF, FKBP1A, KLRB1*, and *FDPS*) were found to be specifically expressed in ECs and were significantly associated with the prognosis of HCC. Pseudotime and cell trajectory analyses of scRNA-seq data indicated that ECs predominantly occupied early stages of the HCC cell differentiation trajectory. These findings suggest that the predictive signature and EC-specific markers identified herein may contribute to the early diagnosis of HCC and serve as potential therapeutic targets. However, while our study provides important insights, there are several limitations we should acknowledge. First, our scRNA-seq analysis included only 12 HCC samples, which may not fully capture the heterogeneity of HCC across different populations or disease stages. Larger and more diverse single-cell datasets will be needed to validate and expand our findings. Second, although we confirmed protein expression of the identified genes in tissue specimens, the molecular mechanisms by which these markers contribute to HCC progression and prognosis remain unclear. Further functional studies are required to clarify their specific roles within ECs and the tumor microenvironment. Third, our prognostic model was constructed and validated using retrospective public datasets (TCGA and ICGC). Although our results were consistent, prospective studies and real-world clinical validation are necessary before clinical application.

## Conclusion

Using scRNA-seq data, we developed and validated an EC-associated five-gene prognostic model for HCC, comprising *NDRG1, HBEGF, FKBP1A, KLRB1*, and *FDPS*. This model demonstrated robust predictive performance for patient outcomes. Experimental validation via immunohistochemistry and Western blotting confirmed the high expression of these proteins in ECs, likely influenced by factors in the HCC tumor microenvironment. Our findings underscore the pivotal role of ECs and their associated markers in the initiation and progression of HCC. These markers may aid in the early detection of HCC and represent promising candidates for future therapeutic development.

## Supporting information

S1 Table The differentiation-related genes (DRGs) for subgroup I. (XLSX)

S2 Table The ECs-related predictive genes in the TCGA cohort. (XLSX)

S1 File Raw images. (PDF)

## References

[1] Sung H, Ferlay J, Siegel RL, Laversanne M, Soerjomataram I, Jemal A, et al. Global cancer statistics 2020: GLOBOCAN estimates of incidence and mortality worldwide for 36 cancers in 185 countries. CA Cancer J Clin. 2021;71(3):209–49. doi: 10.3322/caac.21660 33538338

[2] Siegel RL, Miller KD, Fuchs HE, Jemal A. Cancer statistics, 2022. CA Cancer J Clin. 2022;72(1):7–33. doi: 10.3322/caac.21708 35020204

[3] Reig M, Forner A, Rimola J, Ferrer-Fàbrega J, Burrel M, Garcia-Criado Á, et al. BCLC strategy for prognosis prediction and treatment recommendation: The 2022 update. J Hepatol. 2022;76(3):681–93. doi: 10.1016/j.jhep.2021.11.018 34801630 PMC8866082

[4] Ganesan P, Kulik LM. Hepatocellular Carcinoma: New Developments. Clin Liver Dis. 2023;27(1):85–102. doi: 10.1016/j.cld.2022.08.004 36400469

[5] Johnson PJ. Role of alpha-fetoprotein in the diagnosis and management of hepatocellular carcinoma. J Gastroenterol Hepatol. 1999;14 Suppl:S32-6. doi: 10.1046/j.1440-1746.1999.01873.x 10382636

[6] Halazun KJ, Rosenblatt RE, Mehta N, Lai Q, Hajifathalian K, Gorgen A, et al. Dynamic α-Fetoprotein Response and Outcomes After Liver Transplant for Hepatocellular Carcinoma. JAMA Surg. 2021;156(6):559–67. doi: 10.1001/jamasurg.2021.0954 33950167 PMC8100910

[7] Tyson GL, Duan Z, Kramer JR, Davila JA, Richardson PA, El-Serag HB. Level of α-fetoprotein predicts mortality among patients with hepatitis C-related hepatocellular carcinoma. Clin Gastroenterol Hepatol. 2011;9(11):989–94. doi: 10.1016/j.cgh.2011.07.026 21820396 PMC3200479

[8] Han L-L, Lv Y, Guo H, Ruan Z-P, Nan K-J. Implications of biomarkers in human hepatocellular carcinoma pathogenesis and therapy. World J Gastroenterol. 2014;20(30):10249–61. doi: 10.3748/wjg.v20.i30.10249 25132742 PMC4130833

[9] Chen S, Chen H, Gao S, Qiu S, Zhou H, Yu M, et al. Differential expression of plasma microRNA-125b in hepatitis B virus-related liver diseases and diagnostic potential for hepatitis B virus-induced hepatocellular carcinoma. Hepatol Res. 2017;47(4):312–20. doi: 10.1111/hepr.12739 27152955

[10] Luo P, Wu S, Yu Y, Ming X, Li S, Zuo X, et al. Current Status and Perspective Biomarkers in AFP Negative HCC: Towards Screening for and Diagnosing Hepatocellular Carcinoma at an Earlier Stage. Pathol Oncol Res. 2020;26(2):599–603. doi: 10.1007/s12253-019-00585-5 30661224

[11] Yang ZF, Poon RTP. Vascular changes in hepatocellular carcinoma. Anat Rec (Hoboken). 2008;291(6):721–34. doi: 10.1002/ar.20668 18484619

[12] Kou B, Li Y, Zhang L, Zhu G, Wang X, Li Y, et al. In vivo inhibition of tumor angiogenesis by a soluble VEGFR-2 fragment. Exp Mol Pathol. 2004;76(2):129–37. doi: 10.1016/j.yexmp.2003.10.010 15010291

[13] Wada H, Nagano H, Yamamoto H, Yang Y, Kondo M, Ota H, et al. Expression pattern of angiogenic factors and prognosis after hepatic resection in hepatocellular carcinoma: importance of angiopoietin-2 and hypoxia-induced factor-1 alpha. Liver Int. 2006;26(4):414–23. doi: 10.1111/j.1478-3231.2006.01243.x 16629644

[14] Schoenleber SJ, Kurtz DM, Talwalkar JA, Roberts LR, Gores GJ. Prognostic role of vascular endothelial growth factor in hepatocellular carcinoma: systematic review and meta-analysis. Br J Cancer. 2009;100(9):1385–92. doi: 10.1038/sj.bjc.6605017 19401698 PMC2694418

[15] Poisson J, Lemoinne S, Boulanger C, Durand F, Moreau R, Valla D, et al. Liver sinusoidal endothelial cells: Physiology and role in liver diseases. J Hepatol. 2017;66(1):212–27. doi: 10.1016/j.jhep.2016.07.009 27423426

[16] Shetty S, Lalor PF, Adams DH. Liver sinusoidal endothelial cells - gatekeepers of hepatic immunity. Nat Rev Gastroenterol Hepatol. 2018;15(9):555–67. doi: 10.1038/s41575-018-0020-y 29844586 PMC7096836

[17] Wilkinson AL, Qurashi M, Shetty S. The Role of Sinusoidal Endothelial Cells in the Axis of Inflammation and Cancer Within the Liver. Front Physiol. 2020;11:990. doi: 10.3389/fphys.2020.00990 32982772 PMC7485256

[18] Diehl L, Schurich A, Grochtmann R, Hegenbarth S, Chen L, Knolle PA. Tolerogenic maturation of liver sinusoidal endothelial cells promotes B7-homolog 1-dependent CD8+ T cell tolerance. Hepatology. 2008;47(1):296–305. doi: 10.1002/hep.21965 17975811

[19] Limmer A, Ohl J, Kurts C, Ljunggren HG, Reiss Y, Groettrup M, et al. Efficient presentation of exogenous antigen by liver endothelial cells to CD8+ T cells results in antigen-specific T-cell tolerance. Nat Med. 2000;6(12):1348–54. doi: 10.1038/82161 11100119

[20] Ihling C, Naughton B, Zhang Y, Rolfe PA, Frick-Krieger E, Terracciano LM, et al. Observational Study of PD-L1, TGF-β, and Immune Cell Infiltrates in Hepatocellular Carcinoma. Front Med (Lausanne). 2019;6:15. doi: 10.3389/fmed.2019.00015 30800658 PMC6375852

[21] Su T, Yang Y, Lai S, Jeong J, Jung Y, McConnell M, et al. Single-Cell Transcriptomics Reveals Zone-Specific Alterations of Liver Sinusoidal Endothelial Cells in Cirrhosis. Cell Mol Gastroenterol Hepatol. 2021;11(4):1139–61. doi: 10.1016/j.jcmgh.2020.12.007 33340713 PMC7903131

[22] Zhao Q, Molina-Portela MDP, Parveen A, Adler A, Adler C, E H, et al. Heterogeneity and chimerism of endothelial cells revealed by single-cell transcriptome in orthotopic liver tumors. Angiogenesis. 2020;23(4):581–97. doi: 10.1007/s10456-020-09727-9 32440964 PMC7525283

[23] Lall S, Sinha D, Bandyopadhyay S, Sengupta D. Structure-Aware Principal Component Analysis for Single-Cell RNA-seq Data. J Comput Biol. 2018;:10.1089/cmb.2018.0027. doi: 10.1089/cmb.2018.0027 30133312

[24] Satija R, Farrell JA, Gennert D, Schier AF, Regev A. Spatial reconstruction of single-cell gene expression data. Nat Biotechnol. 2015;33(5):495–502. doi: 10.1038/nbt.3192 25867923 PMC4430369

[25] Qiu X, Mao Q, Tang Y, Wang L, Chawla R, Pliner HA, et al. Reversed graph embedding resolves complex single-cell trajectories. Nat Methods. 2017;14(10):979–82. doi: 10.1038/nmeth.4402 28825705 PMC5764547

[26] Tibshirani R. The lasso method for variable selection in the Cox model. Stat Med. 1997;16(4):385–95. doi: 10.1002/(sici)1097-0258(19970228)16:4<385::aid-sim380>3.0.co;2-3 9044528

[27] Peng C, Gao H, Niu Z, Wang B, Tan Z, Niu W, et al. Integrin αvβ6 and transcriptional factor Ets-1 act as prognostic indicators in colorectal cancer. Cell Biosci. 2014;4(1):53. doi: 10.1186/2045-3701-4-53 25264483 PMC4175281

[28] Kang X-L, He L-R, Chen Y-L, Wang S-B. Role of doublecortin-like kinase 1 and leucine-rich repeat-containing G-protein-coupled receptor 5 in patients with stage II/III colorectal cancer: Cancer progression and prognosis. World J Gastroenterol. 2020;26(43):6853–66. doi: 10.3748/wjg.v26.i43.6853 33268966 PMC7684452

[29] Barry AE, Baldeosingh R, Lamm R, Patel K, Zhang K, Dominguez DA, et al. Hepatic Stellate Cells and Hepatocarcinogenesis. Front Cell Dev Biol. 2020;8:709. doi: 10.3389/fcell.2020.00709 32850829 PMC7419619

[30] Peng H, Zhu E, Zhang Y. Advances of cancer-associated fibroblasts in liver cancer. Biomark Res. 2022;10(1):59. doi: 10.1186/s40364-022-00406-z 35971182 PMC9380339

[31] Kokame K, Kato H, Miyata T. Homocysteine-respondent genes in vascular endothelial cells identified by differential display analysis. GRP78/BiP and novel genes. J Biol Chem. 1996;271(47):29659–65. doi: 10.1074/jbc.271.47.29659 8939898

[32] Wang Y-Y, Zhou Y-Q, Xie J-X, Zhang X, Wang S-C, Li Q, et al. MAOA suppresses the growth of gastric cancer by interacting with NDRG1 and regulating the Warburg effect through the PI3K/AKT/mTOR pathway. Cell Oncol (Dordr). 2023;46(5):1429–44. doi: 10.1007/s13402-023-00821-w 37249744 PMC12974724

[33] Wang J, Lv W, Lin Z, Wang X, Bu J, Su Y. Hsa_circ_0003159 inhibits gastric cancer progression by regulating miR-223-3p/NDRG1 axis. Cancer Cell Int. 2020;20:57. doi: 10.1186/s12935-020-1119-0 32099530 PMC7031989

[34] Bandyopadhyay S, Pai SK, Gross SC, Hirota S, Hosobe S, Miura K, et al. The Drg-1 gene suppresses tumor metastasis in prostate cancer. Cancer Res. 2003;63(8):1731–6. 12702552

[35] Bandyopadhyay S, Pai SK, Hirota S, Hosobe S, Takano Y, Saito K, et al. Role of the putative tumor metastasis suppressor gene Drg-1 in breast cancer progression. Oncogene. 2004;23(33):5675–81. doi: 10.1038/sj.onc.1207734 15184886

[36] Shah MA, Kemeny N, Hummer A, Drobnjak M, Motwani M, Cordon-Cardo C, et al. Drg1 expression in 131 colorectal liver metastases: correlation with clinical variables and patient outcomes. Clin Cancer Res. 2005;11(9):3296–302. doi: 10.1158/1078-0432.CCR-04-2417 15867226

[37] Cangul H. Hypoxia upregulates the expression of the NDRG1 gene leading to its overexpression in various human cancers. BMC Genet. 2004;5:27. doi: 10.1186/1471-2156-5-27 15341671 PMC518960

[38] Wang Z, Wang F, Wang W-Q, Gao Q, Wei W-L, Yang Y, et al. Correlation of N-myc downstream-regulated gene 1 overexpression with progressive growth of colorectal neoplasm. World J Gastroenterol. 2004;10(4):550–4. doi: 10.3748/wjg.v10.i4.550 14966915 PMC4716978

[39] Chua M-S, Sun H, Cheung ST, Mason V, Higgins J, Ross DT, et al. Overexpression of NDRG1 is an indicator of poor prognosis in hepatocellular carcinoma. Mod Pathol. 2007;20(1):76–83. doi: 10.1038/modpathol.3800711 17170744

[40] Akiba J, Ogasawara S, Kawahara A, Nishida N, Sanada S, Moriya F, et al. N-myc downstream regulated gene 1 (NDRG1)/Cap43 enhances portal vein invasion and intrahepatic metastasis in human hepatocellular carcinoma. Oncol Rep. 2008;20(6):1329–35. 19020710

[41] Raab G, Klagsbrun M. Heparin-binding EGF-like growth factor. Biochim Biophys Acta. 1997;1333(3):F179-99. doi: 10.1016/s0304-419x(97)00024-3 9426203

[42] Inui Y, Higashiyama S, Kawata S, Tamura S, Miyagawa J, Taniguchi N, et al. Expression of heparin-binding epidermal growth factor in human hepatocellular carcinoma. Gastroenterology. 1994;107(6):1799–804. doi: 10.1016/0016-5085(94)90823-0 7958694

[43] Tong M, Jiang Y. FK506-Binding Proteins and Their Diverse Functions. Curr Mol Pharmacol. 2015;9(1):48–65. doi: 10.2174/1874467208666150519113541 25986568 PMC6611466

[44] Cai S, Chen Z, Tang H, Meng S, Tao L, Wang Q. Upregulated FKBP1A Suppresses Glioblastoma Cell Growth via Apoptosis Pathway. Int J Mol Sci. 2022;23(23):14935. doi: 10.3390/ijms232314935 36499275 PMC9739687

[45] Li Z, Cui Y, Duan Q, Zhang J, Shao D, Cao X, et al. The Prognostic Significance of FKBP1A and Its Related Immune Infiltration in Liver Hepatocellular Carcinoma. Int J Mol Sci. 2022;23(21):12797. doi: 10.3390/ijms232112797 36361587 PMC9659304

[46] Ye W, Shi Z, Zhou Y, Zhang Z, Zhou Y, Chen B, et al. Autophagy-Related Signatures as Prognostic Indicators for Hepatocellular Carcinoma. Front Oncol. 2022;12:654449. doi: 10.3389/fonc.2022.654449 35402224 PMC8987527

[47] Luo Y, Lin J, Zhang Y, Dai G, Li A, Liu X. LncRNA PCAT6 predicts poor prognosis in hepatocellular carcinoma and promotes proliferation through the regulation of cell cycle arrest and apoptosis. Cell Biochem Funct. 2020;38(7):895–904. doi: 10.1002/cbf.3510 32064636

[48] Rosen DB, Bettadapura J, Alsharifi M, Mathew PA, Warren HS, Lanier LL. Cutting edge: lectin-like transcript-1 is a ligand for the inhibitory human NKR-P1A receptor. J Immunol. 2005;175(12):7796–9. doi: 10.4049/jimmunol.175.12.7796 16339513

[49] Poggi A, Rubartelli A, Moretta L, Zocchi MR. Expression and function of NKRP1A molecule on human monocytes and dendritic cells. Eur J Immunol. 1997;27(11):2965–70. doi: 10.1002/eji.1830271132 9394825

[50] Ma C, Luo H, Cao J, Zheng X, Zhang J, Zhang Y, et al. Identification of a novel tumor microenvironment-associated eight-gene signature for prognosis prediction in lung adenocarcinoma. Front Mol Biosci. 2020;7:571641. doi: 10.3389/fmolb.2020.571641 33102522 PMC7546815

[51] Xiang S, Li J, Shen J, Zhao Y, Wu X, Li M, et al. Identification of Prognostic Genes in the Tumor Microenvironment of Hepatocellular Carcinoma. Front Immunol. 2021;12:653836. doi: 10.3389/fimmu.2021.653836 33897701 PMC8059369

[52] Bao X, Shi R, Zhang K, Xin S, Li X, Zhao Y, et al. Immune Landscape of Invasive Ductal Carcinoma Tumor Microenvironment Identifies a Prognostic and Immunotherapeutically Relevant Gene Signature. Front Oncol. 2019;9:903. doi: 10.3389/fonc.2019.00903 31620363 PMC6759595

[53] Cheng X, Cao Y, Wang X, Cheng L, Liu Y, Lei J, et al. Systematic Pan-Cancer Analysis of KLRB1 with Prognostic Value and Immunological Activity across Human Tumors. J Immunol Res. 2022;2022:5254911. doi: 10.1155/2022/5254911 35028320 PMC8749375

[54] Sun Y, Wu L, Zhong Y, Zhou K, Hou Y, Wang Z, et al. Single-cell landscape of the ecosystem in early-relapse hepatocellular carcinoma. Cell. 2021;184(2):404-421.e16. doi: 10.1016/j.cell.2020.11.041 33357445

[55] Seshacharyulu P, Rachagani S, Muniyan S, Siddiqui JA, Cruz E, Sharma S, et al. FDPS cooperates with PTEN loss to promote prostate cancer progression through modulation of small GTPases/AKT axis. Oncogene. 2019;38(26):5265–80. doi: 10.1038/s41388-019-0791-9 30914801 PMC6597298

[56] Abate M, Laezza C, Pisanti S, Torelli G, Seneca V, Catapano G, et al. Deregulated expression and activity of Farnesyl Diphosphate Synthase (FDPS) in Glioblastoma. Sci Rep. 2017;7(1):14123. doi: 10.1038/s41598-017-14495-6 29075041 PMC5658376

[57] Notarnicola M, Messa C, Cavallini A, Bifulco M, Tecce MF, Eletto D, et al. Higher farnesyl diphosphate synthase activity in human colorectal cancer inhibition of cellular apoptosis. Oncology. 2004;67(5–6):351–8. doi: 10.1159/000082918 15713990

[58] Seshacharyulu P, Halder S, Nimmakayala R, Rachagani S, Chaudhary S, Atri P, et al. Disruption of FDPS/Rac1 axis radiosensitizes pancreatic ductal adenocarcinoma by attenuating DNA damage response and immunosuppressive signalling. EBioMedicine. 2022;75:103772. doi: 10.1016/j.ebiom.2021.103772 34971971 PMC8718746

[59] Tang L, Wei R, Chen R, Fan G, Zhou J, Qi Z, et al. Establishment and validation of a cholesterol metabolism-related prognostic signature for hepatocellular carcinoma. Comput Struct Biotechnol J. 2022;20:4402–14. doi: 10.1016/j.csbj.2022.07.030 36051877 PMC9420502

[60] Hassani SF, Sayaf M, Danandeh SS, Nourollahzadeh Z, Shahmohammadi M, Akbari S, et al. Novel Insight Into the Association Between Obesity and Hepatocellular Carcinoma Occurrence and Recurrence: High-Throughput Microarray Data Set Analysis of Differentially Expressed Genes. JCO Clin Cancer Inform. 2021;5:1169–80. doi: 10.1200/CCI.21.00094 34860577

