# Supplementary material for: Role of endothelial cell markers in prognosis of hepatocellular carcinoma: Integrating bioinformatics analysis and experimental validation
Source: PLoS One. 2025 Sep 16;20(9):e0331580. doi: 10.1371/journal.pone.0331580 (PMC12440173; doi:10.1371/journal.pone.0331580)

NDRG1 original figure

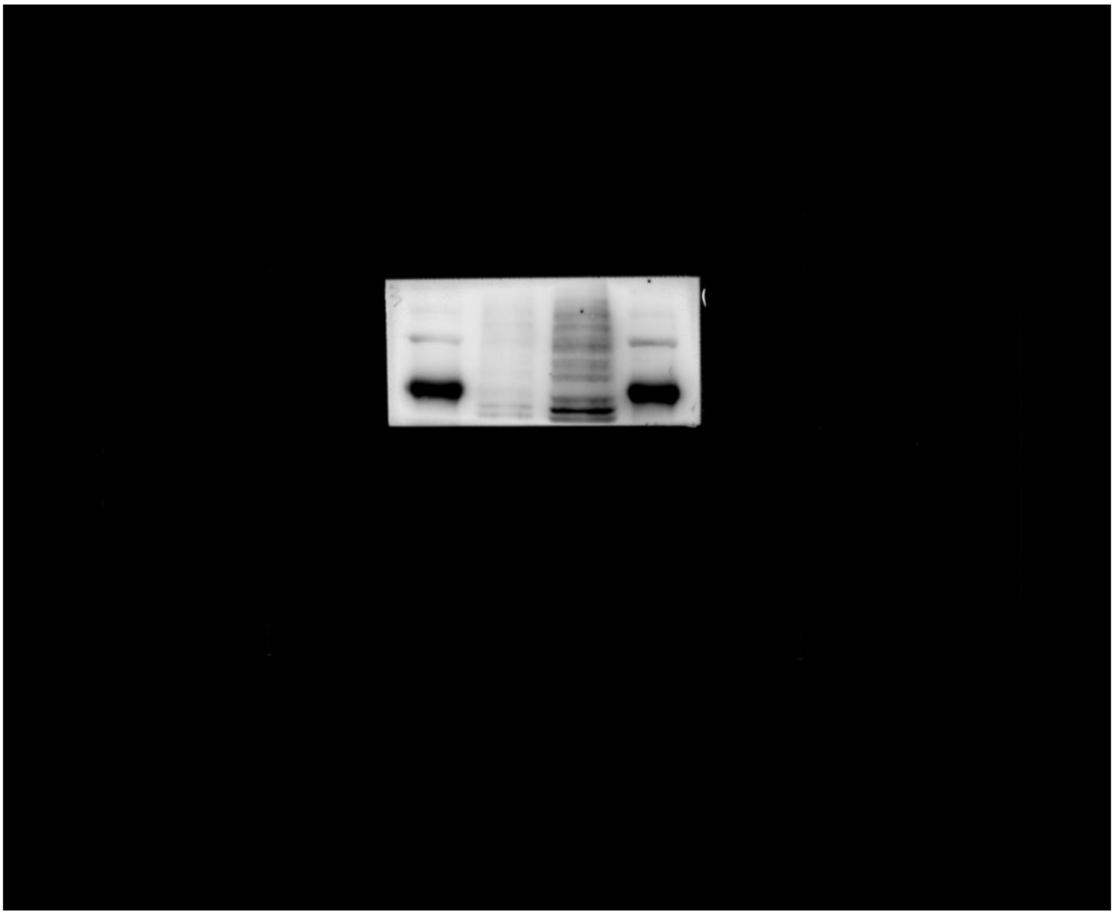

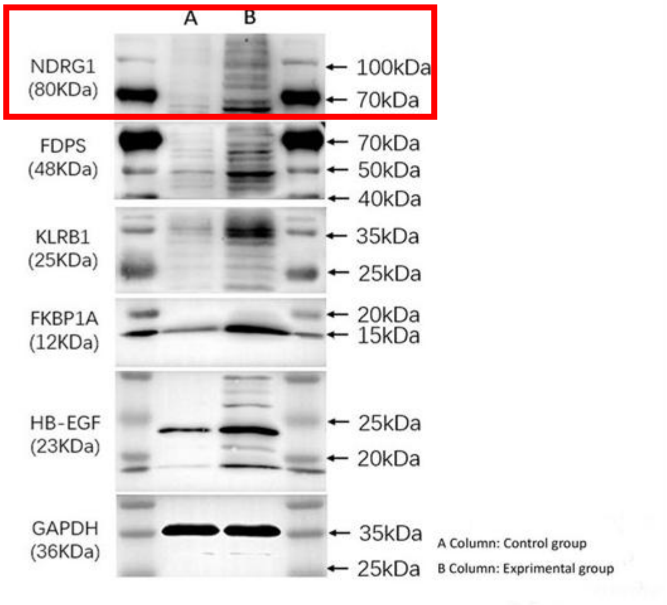

FDPS original figure

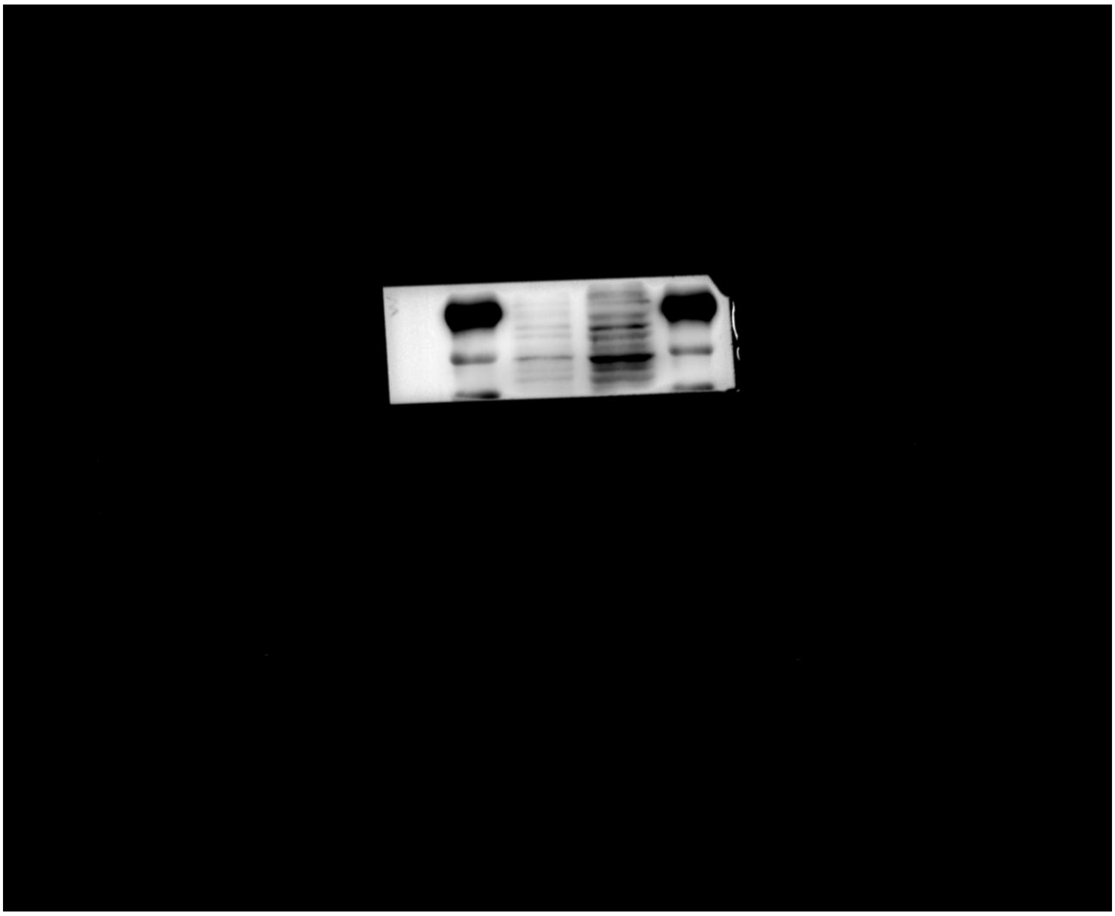

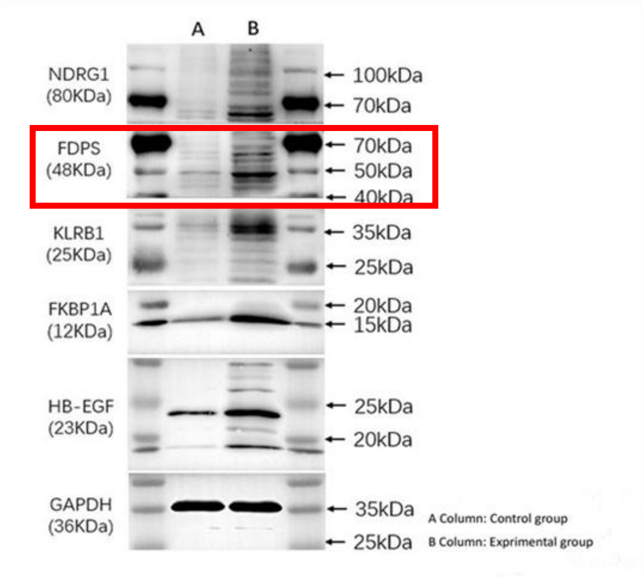

KLRB1 original figure

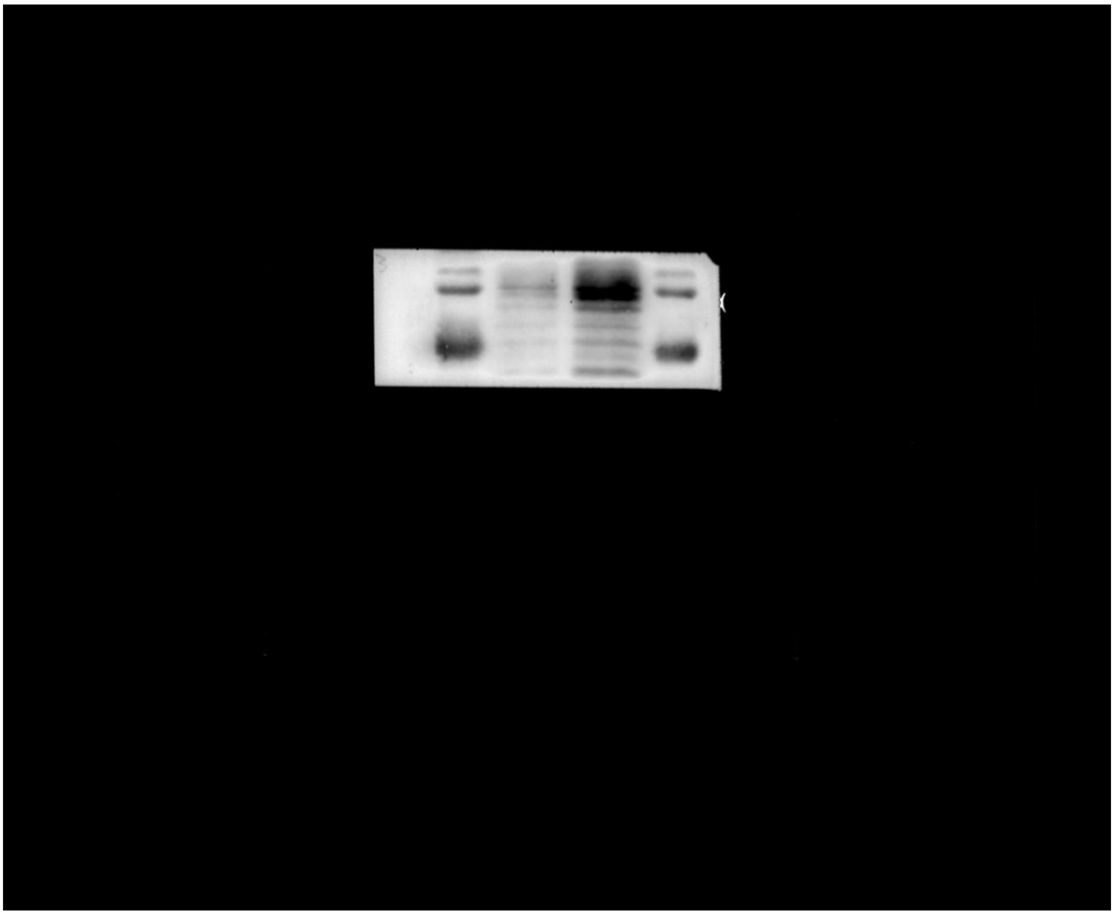

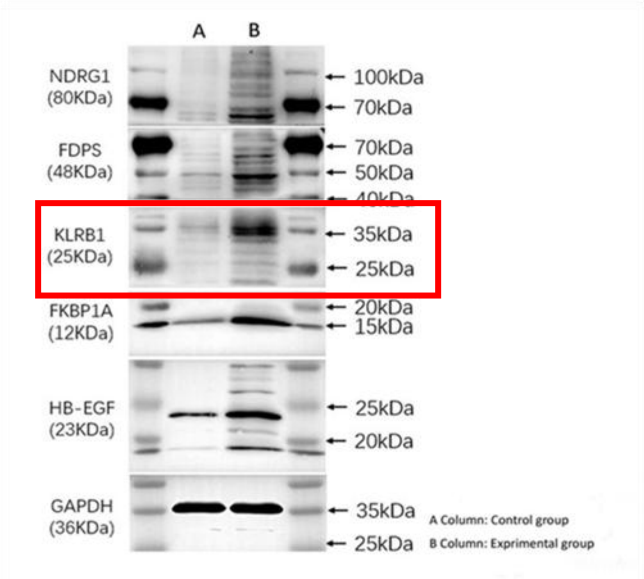

FKBP1A original figure

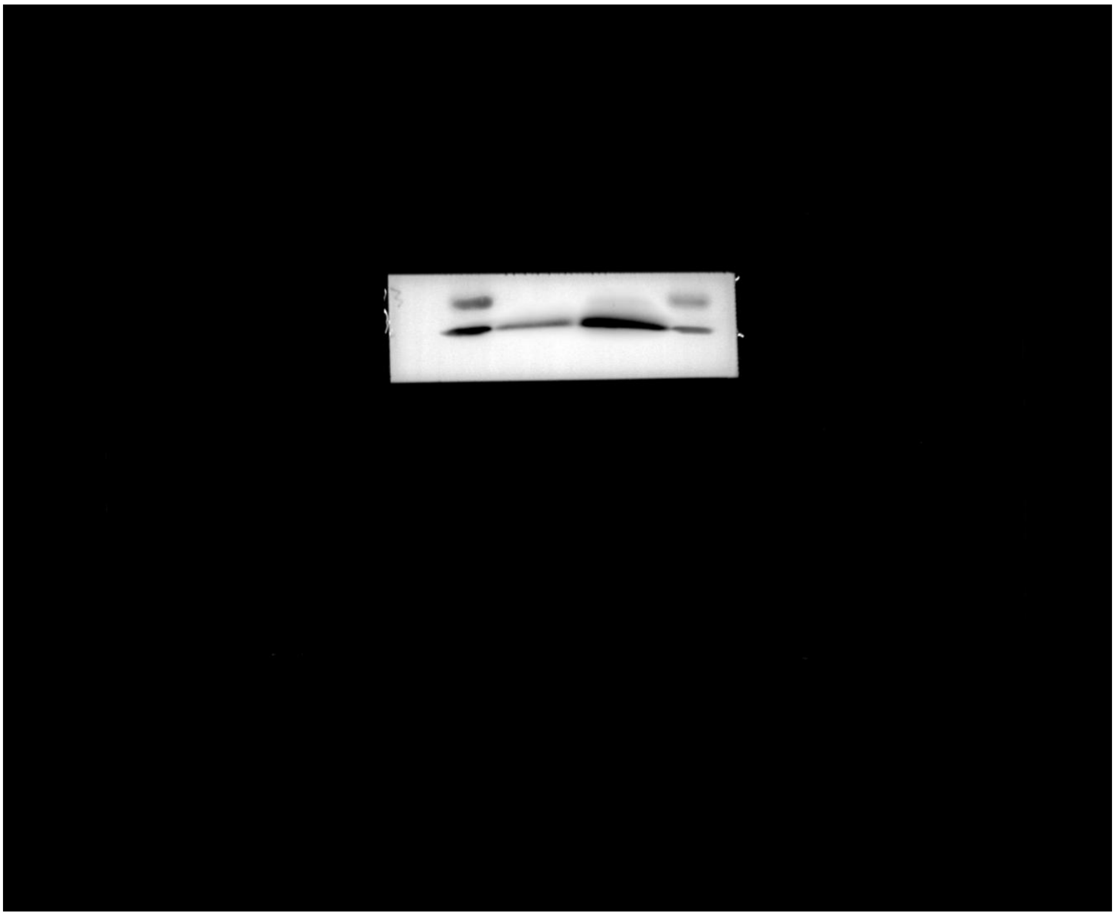

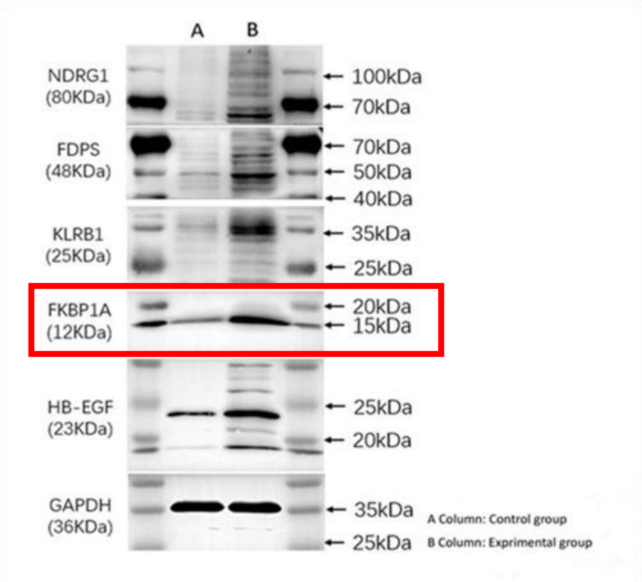

HB-EGF original figure

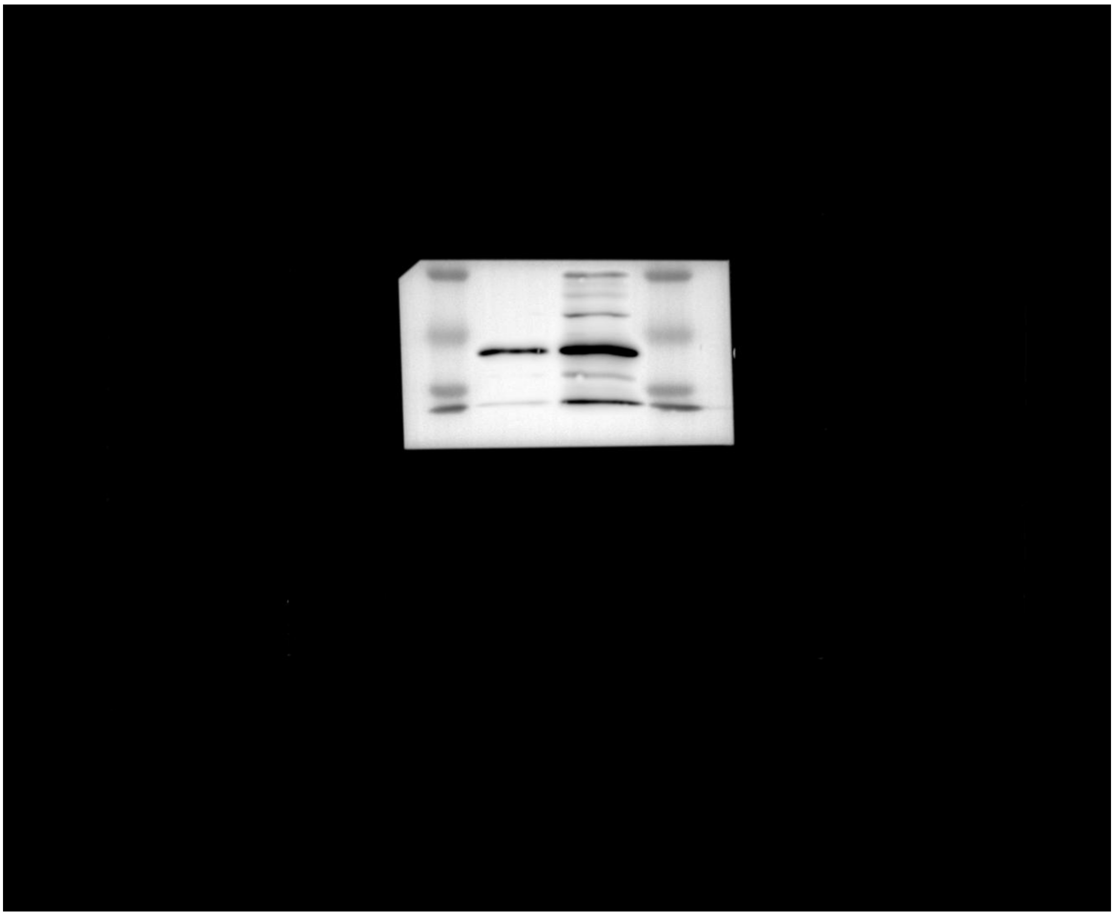

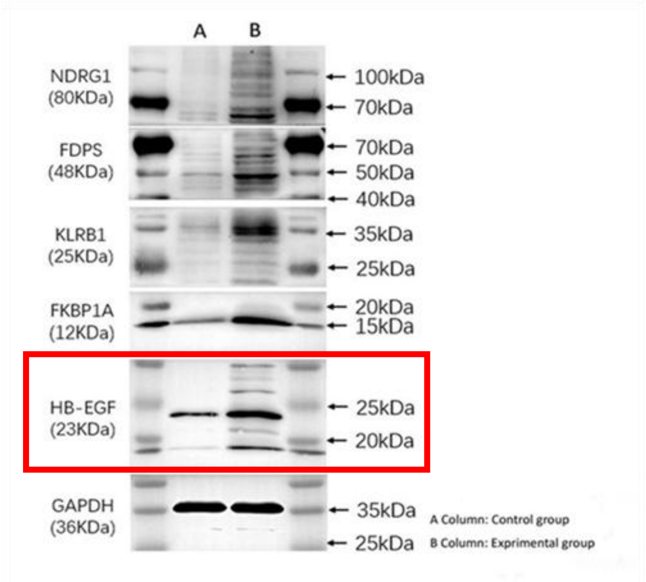

GAPDH original figure

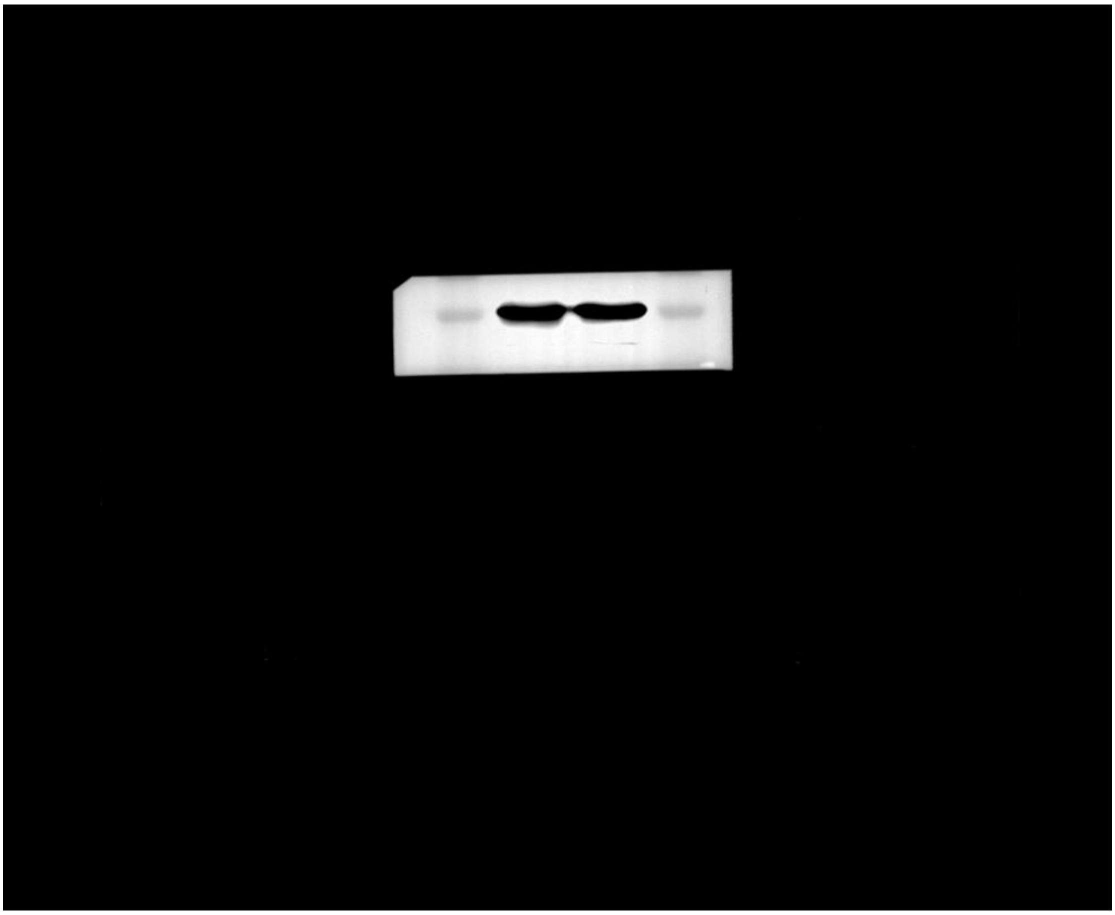

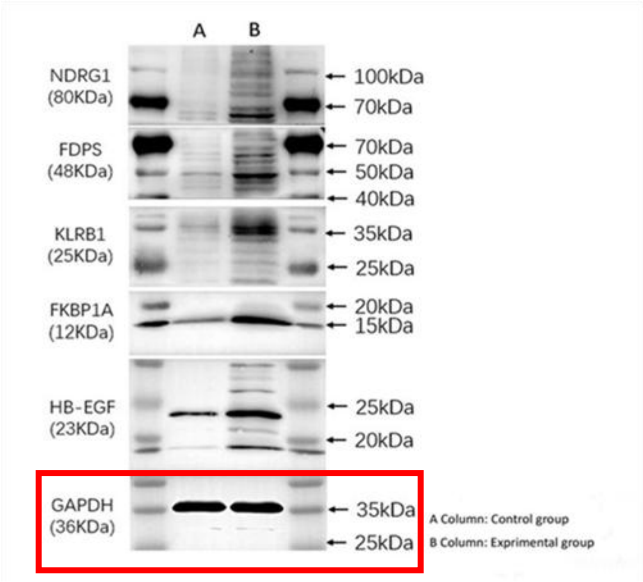

Supplement: S1 File — (PDF) [file pone.0331580.s003.pdf]
